# GDF15 attenuates myocardial infarction-induced injury by preserving mitochondrial function and suppressing oxidative stress

**DOI:** 10.1186/s40001-025-03144-8

**Published:** 2025-09-29

**Authors:** Xiaogang Yuan, Cheng Wang, Haiyan Zhu

**Affiliations:** 1 Department of Critical Care Medicine, the First Affiliated Hospital, Jiangxi Medical College, Nanchang University, Nanchang, China; 2 The Second Affiliated Hospital, School of Medicine, The Chinese University of Hong Kong, Shenzhen & Longgang District People’s Hospital of Shenzhen, Shenzhen, China; 3 School of Pharmaceutical Sciences, Anhui Medical University, Hefei, China; 4 The First Clinical Medical College of Nanchang University, Nanchang, Jiangxi China; 5 Department of Emergency, First Medical Center of Chinese PLA General Hospital, Beijing, China

**Keywords:** GDF15, Myocardial infarction, Mitochondrial function, Oxidative stress

## Abstract

Myocardial infarction, a serious cardiovascular disease, is still a major cause of morbidity and mortality worldwide. Growth differentiation factor-15, a stress-responsive cytokine, has been involved in cardiac pathophysiology, but its exact role in myocardial infarction remains controversial. This study aimed to clarify the mechanisms underlying the cardioprotective effects of GDF-15 in myocardial infarction. By using a combination of in vivo and in vitro methods, including immunofluorescence staining, echocardiography, RNA sequencing, and high-resolution respirometry, we showed that GDF-15 expression is significantly upregulated in infarcted myocardium and its deficiency aggravates cardiac injury. Mechanistically, GDF-15 deficiency impairs mitochondrial function and energy metabolism under hypoxic stress, as evidenced by changes in mitochondrial membrane potential and respiratory parameters. Moreover, we identified that GDF-15 suppresses hypoxia-induced reactive oxygen species generation through activation of the AMPK signaling pathway. Therapeutic administration of exogenous GDF-15 reduces myocardial injury, hypoxic stress, and fibrosis after myocardial infarction, suggesting its potential as a therapeutic target. These findings collectively demonstrate that GDF-15 plays a crucial role in cardiac protection during myocardial infarction by regulating mitochondrial function, energy metabolism, and oxidative stress. Our results provide novel insights into the molecular mechanisms of GDF-15-mediated cardioprotection and suggest its potential as a therapeutic intervention for myocardial infarction. Future studies should focus on translational research to evaluate the clinical efficacy of GDF-15-based therapies in myocardial infarction patients.

**Supplementary Information:**

The online version contains supplementary material available at 10.1186/s40001-025-03144-8.

## Introduction

Myocardial infarction (MI), a leading cause of morbidity and mortality worldwide, is characterized by cardiomyocyte death resulting from prolonged ischemia [1–3]. Despite advances in reperfusion therapies, the complex pathophysiological mechanisms underlying MI onset and subsequent cardiac remodeling remain incompletely understood [4–6].

Growth differentiation factor 15 (GDF15), a member of the transforming growth factor-beta superfamily, participates in diverse physiological and pathological processes including inflammation and cancer [7–9]. GDF15 acts as a stress-responsive cytokine that signals through the glial cell line-derived neurotrophic factor (GDNF) family receptor α-like (GFRAL) complexed with receptor tyrosine kinase RET, predominantly expressed in the brainstem area postrema [10]. This GFRAL-RET signaling axis regulates energy homeostasis, food intake, and metabolic responses to cellular stress [11, 12]. In cardiovascular pathologies, elevated GDF15 levels occur in acute coronary syndromes, heart failure, and atherosclerosis, correlating with disease severity and outcomes [13, 14]. Beyond cardiac conditions, GDF15 is upregulated in metabolic disorders, chronic kidney disease, and inflammatory states, functioning both as a protective factor during acute stress and a potential mediator of chronic pathological processes [15]. However, its specific role in MI remains inadequately characterized. Current GDF15 research has primarily addressed its functions in cancer and inflammatory diseases, where it modulates immune responses and cell survival [16].

In the MI context, few studies have investigated GDF15 expression and functional implications [17–19]. Preliminary evidence suggests GDF15 may exert cardioprotective effects, potentially through anti-inflammatory and anti-fibrotic properties [20–22]. Nevertheless, the precise mechanisms by which GDF15 influences MI progression and cardiac remodeling remain poorly defined.

This study aims to elucidate the regulatory mechanisms and functional roles of GDF15 in MI. By providing insights into GDF15’s protective effects and its downstream pathways, our research seeks to contribute to the development of novel therapeutic strategies. The integration of molecular, cellular, and in vivo analyses highlights GDF15 as a promising candidate for future clinical interventions in cardiac disease.

## Materials and methods

### Reagent

CoCl2·6H2O was obtained from Good Laboratory Practice Bioscience (Cat. No. GB57786). Antimycin A (Cat. No. MS0070-10MG) and Oligomycin (Cat. No. MZ8001-5MG) were purchased from Maokang Biotechnology. Carbonyl cyanide m-chlorophenylhydrazone (CCCP, Cat. No. C2759) and Rotenone (Rot, Cat. No. R8775) were acquired from Sigma-Aldrich. DMEM medium (Cat. No. C11995500BT) and fetal bovine serum (FBS, Cat. No. 10270–106) were sourced from Gibco. Penicillin/streptomycin solution (Cat. No. HY-K1006) was obtained from MedChemExpress. Dorsomorphin (Catalog No. S7840) was purchased from Selleck Chemicals (Houston, TX, USA).

### Generation of GDF15 knockout mice

Male wild-type (WT) and *GDF15* knockout (KO) mice were used in this study. *GDF15* KO mice were generated using CRISPR/Cas9 technology, targeting exon 2 of the GDF15-201 transcript which encodes most of the coding sequence. In vitro transcribed gRNA along with Cas9 protein was microinjected into fertilized eggs from C57BL/6JGpt mice. The injected eggs were transplanted to obtain F0 generation mice, validated by PCR and sequencing analysis. Stable F1 generation knockout mice were established by mating PCR-positive F0 founders with wildtype C57BL/6JGpt mice. Additionally, *GDF15* KO mice on the C57BL/6N background (C57BL/6N-Gdf15tm1a(KOMP)Wtsi/H) were obtained from the International Mouse Phenotyping Consortium. To minimize genetic variability, all experiments were performed using age-matched littermate controls generated from heterozygous breeding pairs. Genotyping was performed by PCR using tail DNA according to protocols established by the IMPC.

### Cell culture and treatment

Human AC16 cardiomyocytes were maintained in DMEM supplemented with 10% FBS and 1% penicillin/streptomycin at 37 °C in a humidified atmosphere containing 5% CO2. Primary cardiomyocytes were isolated from 0 to 3-day-old Sprague–Dawley rats using the Primary Cardiomyocyte Isolation Kit (Cat. No. 88281, Thermo Fisher Scientific) according to manufacturer’s instructions. Briefly, isolated ventricles were placed in pre-chilled HBSS and minced into 1–3 mm^2^ pieces. The tissue fragments were enzymatically digested with a mixture of Enzyme 1 and Enzyme 2 (20:1) at 37 °C for 30 min under 5% CO2. After washing with HBSS, cells were pre-plated in 15-cm dishes containing DMEM supplemented with 10% FBS and 1% penicillin/streptomycin for 90 min at 37 °C to remove non-myocardial cells. The non-adherent cardiomyocytes were collected and seeded into NEST confocal dishes. For hypoxia experiments, cells were treated with 250 μM CoCl2 and/or 100 ng/ml recombinant GDF15 (Cat. No. GD5-M5149, ACROBiosystems).

### Animal experiments

All experiments used 8–10-week-old male C57BL/6 or GDF15 knockout (*GDF15* KO) mice from Jiangsu GemPharmatech Co., Ltd., which were randomly placed in cages. A permanent myocardial infarction (MI) model was established by ligation of the left anterior descending (LAD) coronary artery. During the surgical procedure, Alzet Osmotic Pumps (# 1003D, Alzet) were implanted subcutaneously to deliver either GDF15 or saline at a rate of 12 μg/day. Briefly, the mice were anesthetized with 2% isoflurane, intubated, and the chest area was shaved. Perioperative pain management included buprenorphine (0.1 mg/kg SC, 30 min pre-surgery and q12h for 72 h) and meloxicam (5 mg/kg SC daily for 3d), with isoflurane (1.5–2%) for anesthesia and topical lidocaine (0.5%) at the incision site. Animals were monitored daily for pain indicators, with additional analgesics administered as needed according to veterinary guidance. The thoracic cavity was reached through an incision between the fourth and fifth intercostal spaces and fixed with a chest retractor. The LAD was carefully identified and permanently occluded using a 7–0 prolene suture. Successful establishment of MI was confirmed by the visible paling of the anterior ventricular wall distal to the suture and ST-segment elevation on the electrocardiogram (ECG). After thoracic closure, the mice were extubated and placed on a heating pad to recover. During the process, the ventilator parameters were kept at: inspiration/expiration ratio 1:1, respiratory rate 90–100 breaths/minute, and tidal volume 1–2 mL. For all interventional studies, tissue collection was performed 7 days post-myocardial infarction. Due to the inherent biological variability in the surgical MI model, experiments were conducted in distinct batches. Animals that did not survive the surgical procedure were not included in the analysis. All experiments in this study adhered to ARRIVE guidelines and received approval from the professional ethics committee (2024088DW).

### Exogenous GDF15 administration

Recombinant GDF15 protein was administered via subcutaneous osmotic pumps. Mice were randomly assigned to receive either saline (control) or GDF15 (12 μg/kg). The GDF15 solution was prepared at 520.8 ng/μl, with each pump containing 110 μl (total GDF15: 0.286 mg dissolved in saline). Pumps were implanted immediately following MI surgery, and treatment was maintained throughout the study duration.

### Echocardiography

Transthoracic echocardiography was performed using a VINNO 6 LAB ultrasound system with X10-23L probe. Mice were prepared by removing fur from the chest area prior to imaging. Animals were anesthetized in an induction chamber and immobilized on the platform with adhesive tape. Ultrasound coupling gel was applied to the chest for cardiac assessment. All echocardiographic measurements were performed at standardized mid-papillary level using consistent anatomical landmarks across experimental groups to ensure reproducibility. Left ventricular function was evaluated before surgery and post-MI. Parameters included ejection fraction and fractional shortening, with measurements recorded and analyzed by the same blinded operator.

### Measurement of intracellular reactive oxygen species (ROS) levels

Intracellular ROS levels were evaluated using a ROS Detection Kit. DCFH-DA and Hoechst 33342 were diluted in serum-free medium and incubated with AC16 cells or primary cardiomyocytes for 15 min at 37 °C in darkness. Images were captured using fluorescence microscopy.

### Mitochondrial membrane potential assay

The mitochondrial membrane potential (MMP) was evaluated using the JC-1 dye, a ratiometric fluorescent probe that indicates mitochondrial health (Sivandzade et al., 2019). After the indicated treatments, AC16 cells were incubated with JC-1 staining solution (5 µg/mL; cat. no. C2006, Beyotime, China) for 20 min at 37 °C in the dark, according to the manufacturer’s protocol. The cells were subsequently washed twice with ice-cold JC-1 staining buffer. Images were captured immediately using a fluorescence microscope (Olympus, Japan). In healthy mitochondria with high membrane potential, JC-1 forms J-aggregates that emit red fluorescence. Conversely, in unhealthy cells with low membrane potential, JC-1 remains in its monomeric form, emitting green fluorescence. The change in MMP was quantified by calculating the ratio of red to green fluorescence intensity. A decrease in this ratio indicates mitochondrial depolarization.

### Western blot analysis

Cellular proteins were extracted using RIPA buffer supplemented with phosphatase and protease inhibitors. Protein concentrations were determined using a BCA protein assay kit. Equal amounts of protein were separated by 8–12.5% SDS-PAGE and transferred to nitrocellulose membranes. Membranes were blocked with TBS containing 0.1% Tween 20 and 5% non-fat milk for 1 h at room temperature. Primary antibody incubation was performed overnight at 4 °C, followed by three 5-min washes with TBST. Horseradish peroxidase-conjugated secondary antibodies were applied for protein detection.

### Histological analysis

Mouse cardiac tissues were fixed in 10% neutral buffered formalin overnight, dehydrated through a graded ethanol series, and embedded in paraffin. Tissue sections were prepared using a microtome and stained with hematoxylin and eosin and Masson’s trichrome following standard protocols.

### Immunofluorescence analysis

Paraffin sections were deparaffinized at 70 °C and rehydrated through xylene and graded ethanol series (100%, 95%, 85%). After PBS washing, sections were treated with 3% hydrogen peroxide for 8 min. Antigen retrieval was performed using citrate buffer. Sections were permeabilized with 0.3% Triton X-100 and blocked with 3% BSA for 1 h. Primary antibodies against HIF-1α (1:200, Cat. No. sc-13515, Santa Cruz Biotechnology) or GDF15 (1:150, Cat. No. sc-515675, Santa Cruz Biotechnology) were applied overnight at 4 °C, followed by incubation with fluorophore-conjugated goat anti-mouse IgG secondary antibody (1:500, Cat. No. A11001, Thermo Fisher Scientific) for 1 h at 37 °C. Nuclei were counterstained with DAPI (Cat. No. P0131-25 ml, Beyotime) for 5 min at room temperature.

### O2K analysis

Oxygen consumption was measured using an Oroboros Oxygraph-O2k (Oroboros Instruments GmbH, Innsbruck, Austria). Oxygen concentration was set to 200 nmol O2/ml in each chamber, and data were recorded every 2 s using DatLab 5 software. AC16 cells were harvested by trypsinization, resuspended in MiR05 respiratory medium, and 1 × 10^6 cells were added to each chamber containing 2 ml of MiR05. Sequential additions of oligomycin (5 mM/chamber), CCCP (0.5 μM/chamber), rotenone (0.5 μM/chamber), and antimycin A (2.5 μM/chamber) were made to assess different respiratory states.

### Statistical analysis

Data are presented as mean ± standard error of the mean (SEM). Statistical comparisons between two groups were performed using unpaired two-tailed Student’s t-tests. For multiple group comparisons, one-way analysis of variance (ANOVA) followed by Fisher’s least significant difference (LSD) post hoc test was employed. P values < 0.05 were considered statistically significant. All statistical analyses were conducted using GraphPad Prism software (version 8.0, GraphPad Software Inc., San Diego, CA, USA).

## Results

### GDF15 expression is upregulated in myocardial infarction and its deficiency exacerbates cardiac injury

**Fig. 1:**
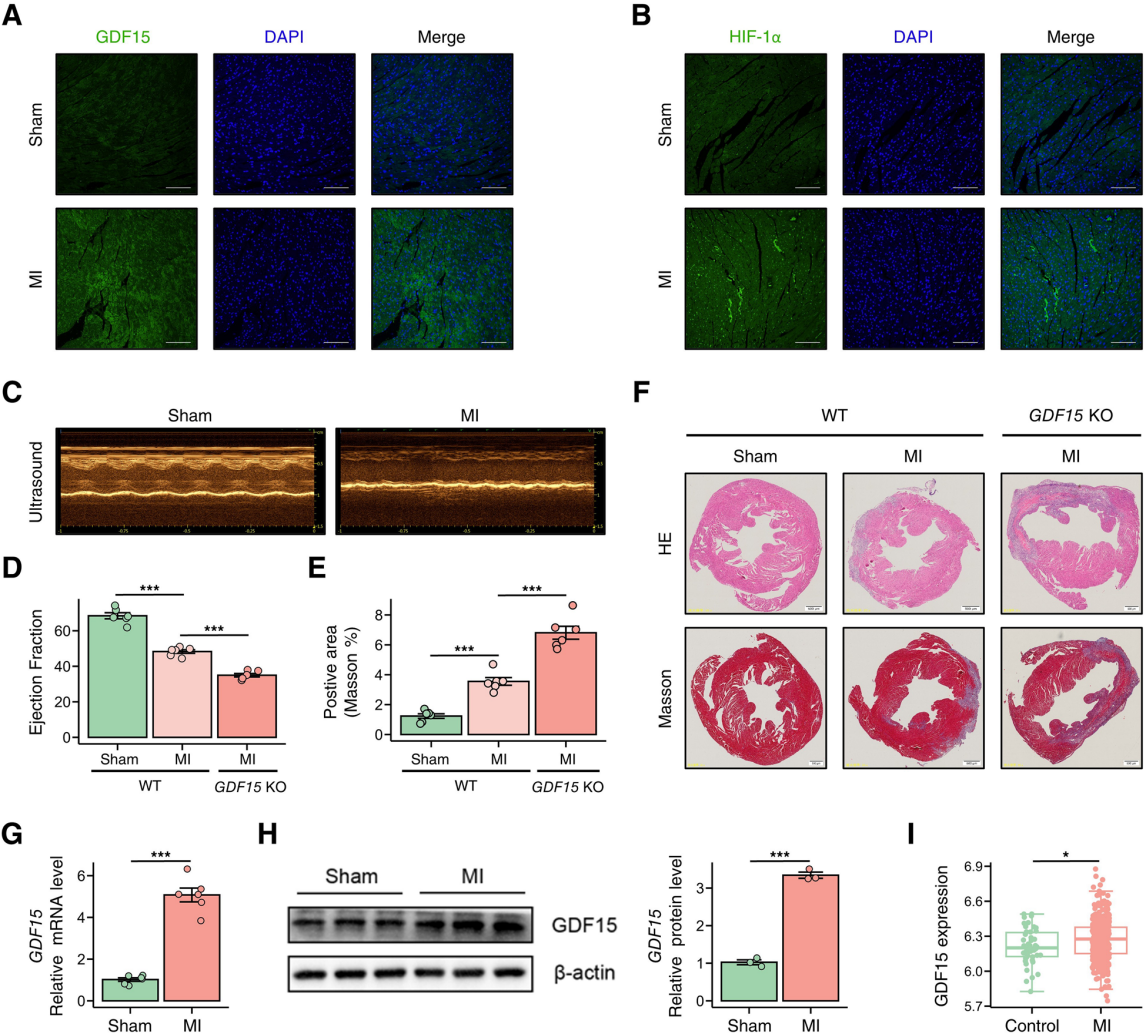
GDF15 increased in MI mouse, and deficiency of GDF15 alleviate the fibrosis. **A**–**B** Representative immunofluorescence images showing GDF15 and HIF-1α expression in heart tissues from Sham and MI mice. **C**–**D** Representative echocardiographic images of hearts from different experimental groups, with quantification of ejection fraction. **E**–**F** GDF15 deficiency attenuated myocardial inflammation and fibrosis following MI, with quantification of positive area. **G**–**H** MI surgery induced upregulation of GDF15 expression in mouse cardiac tissues. **I** GDF15 expression was increased in peripheral blood from MI patients. Data are presented as mean ± SD. n = 6–8/group. *p* < 0.05, *p* < 0.001 by two-tailed Student’s t test or one-way ANOVA. Scale bar, 200 μm. anti-GDF-15 (1:1000, Abcam); anti-HIF-1α (1:1000, Abcam)

To investigate the role of GDF15 in myocardial infarction (MI), we initially examined its expression pattern in heart tissues from MI mice. Immunofluorescence analysis revealed marked upregulation of both GDF15 and HIF-1α in infarcted myocardium compared to sham-operated controls (Fig. 1A–B). Echocardiographic assessment demonstrated characteristic alterations in cardiac structure and function across experimental groups (Fig. 1C–D and Table S1). Both mRNA and protein expression of GDF15 were upregulated following MI (Fig. 1G–H). Notably, GDF15 deficiency significantly exacerbated myocardial inflammation and fibrosis following MI (Fig. 1E–F and Fig S1). Analysis of a previously published dataset (GSE59867) revealed significantly elevated GDF15 expression in peripheral blood of MI patients (Fig. 1I), suggesting a critical role in mediating post-infarction cardiac remodeling.

### GDF15 deficiency impairs mitochondrial function and energy metabolism under hypoxic stress

**Fig. 2:**
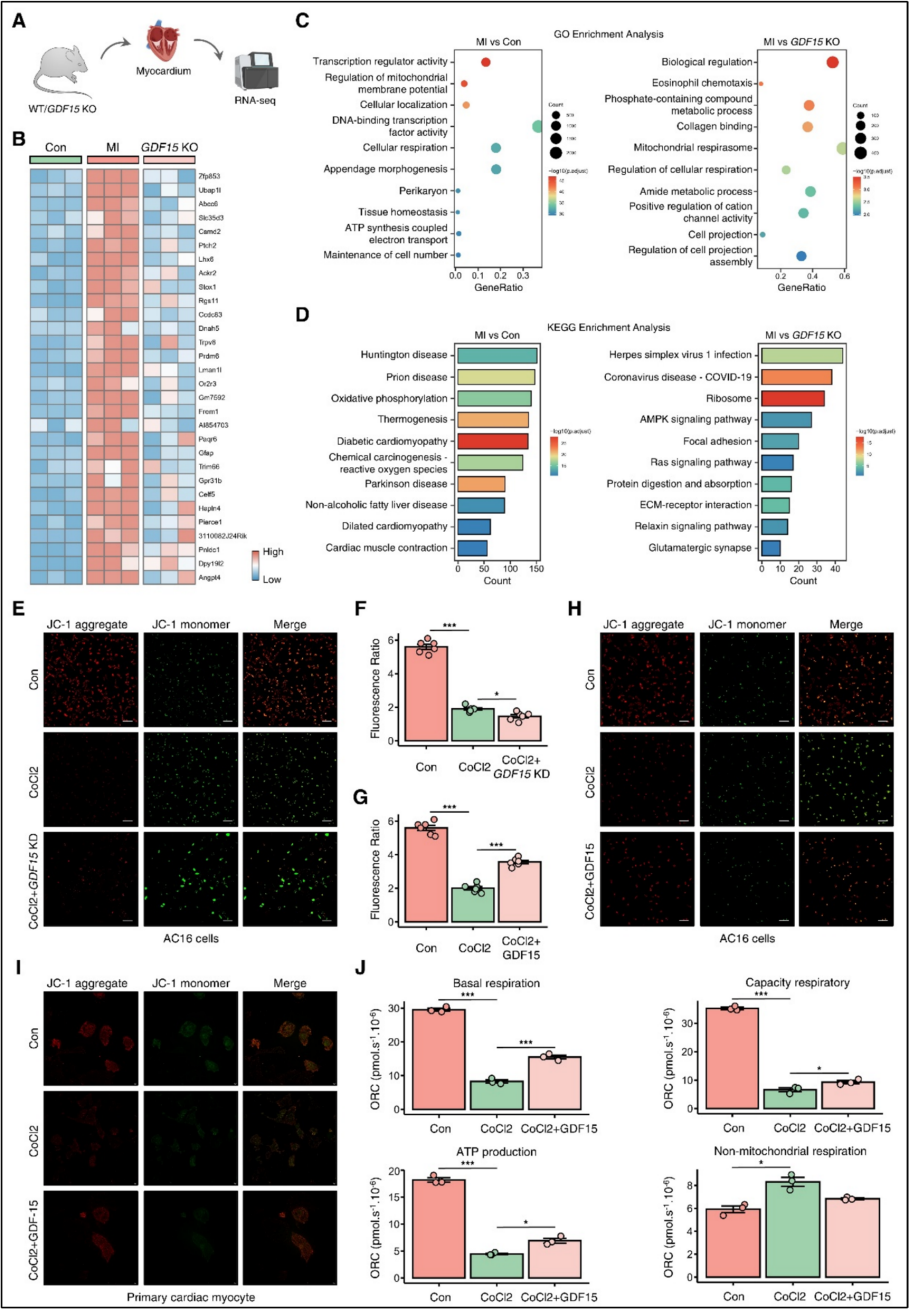
GDF15 regulated metabolic reprogramming. **A**–**B** RNA-seq of myocardial tissue from WT and *GDF15* KO mice. Using the DEGs of RNA-seq profile to GO enrichment (**C**) and KEGG enrichment (**D**). **E**–**H** Immunofluorescence showed the membrane potential of AC16 cells under different treatment. **I** Immunofluorescence showed the Membrane potential of primary cardiac myocyte under different treatment. **J** Basal res0piration, capacity respiratory, ATP production, and non-mitochondrial respiration in AC16 cells. Data are presented as mean ± SD. *p* < 0.05, *p* < 0.001 by two-tailed Student’s t test or one-way ANOVA. Scale bar, 200 μm

To elucidate molecular mechanisms underlying GDF15-mediated cardioprotection, we performed RNA sequencing comparing left ventricular specimens from wild-type and GDF15 knockout mice (n = 6–8 per group) harvested 7 days post-MI (Fig. 2A). Figure 2B depicts the most significantly differentially expressed genes (DEGs) across experimental groups. Gene Ontology and KEGG pathway enrichment analyses of these DEGs revealed significant enrichment of terms related to mitochondrial function and cellular respiration, suggesting their involvement in GDF15-mediated cardioprotection (Fig. 2C–D). To further investigate GDF15’s metabolic effects, we assessed mitochondrial membrane potential in both AC16 cardiomyocytes and primary cardiac myocytes under various treatment conditions. Immunofluorescence analysis demonstrated significant alterations in membrane potential in AC16 cells across treatment groups (Fig. 2E–H), with comparable effects observed in primary cardiac myocytes (Fig. 2I, p < 0.05). High-resolution respirometry revealed that GDF15 significantly influenced key parameters of mitochondrial function, including basal respiration, respiratory capacity, ATP production, and non-mitochondrial respiration in AC16 cells (Fig. 2J, p < 0.05).

### GDF15 suppresses hypoxia-induced ROS generation through AMPK signaling

**Fig. 3:**
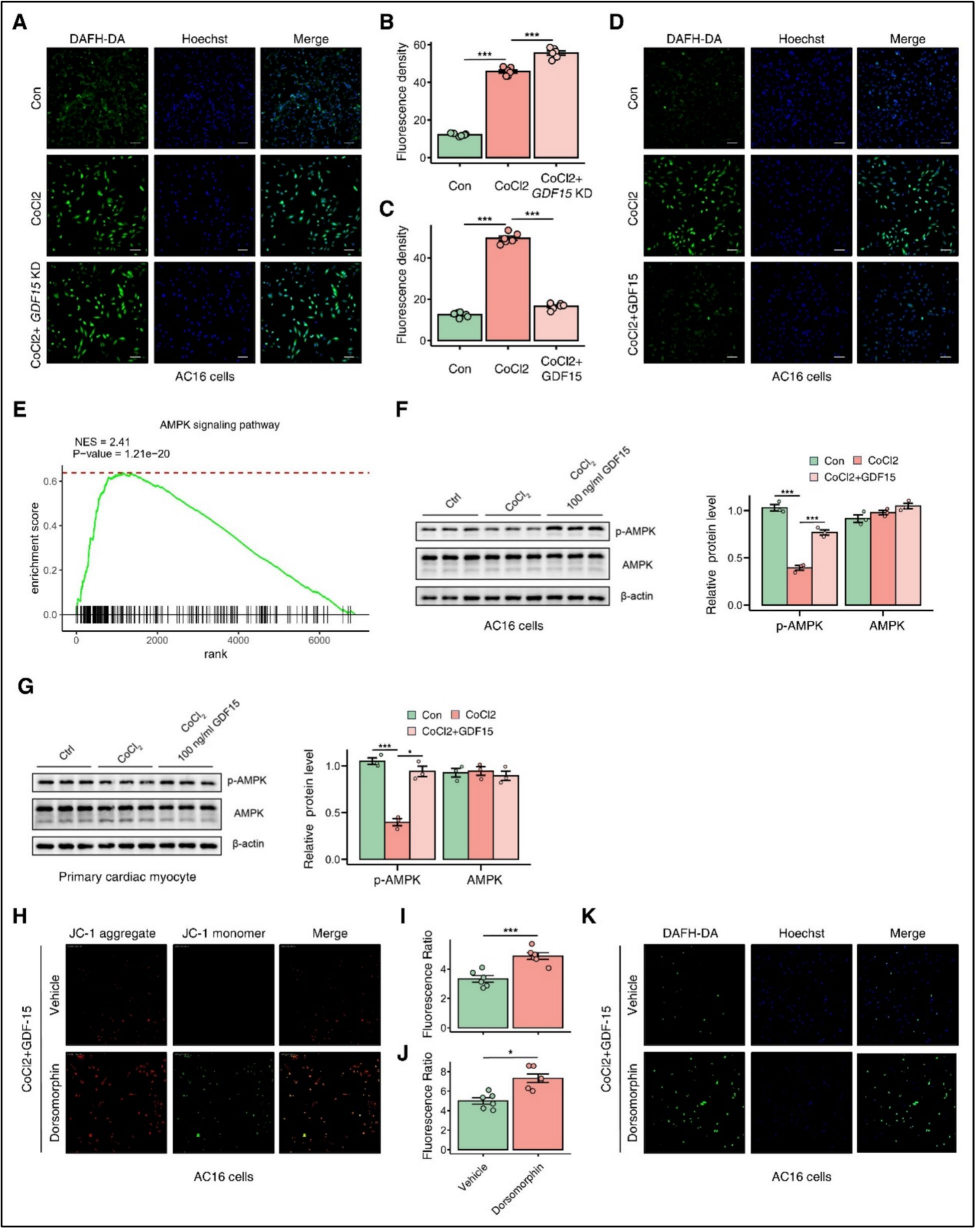
GDF15 inhibited ROS production through AMPK signaling. **A**–**D** ROS production in AC16 cells under different treatment. **E** GSEA of RNA-seq profile from WT and *GDF15* KO mice. **F**–**G** Protein expression of p-AMPK and AMPK in AC16 cells and primary cardiac myocyte. **H**–**I** Immunofluorescence showed the membrane potential of AC16 cells under different treatment. **J**–**K** ROS production in AC16 cells under different treatment. Data are presented as mean ± SD. *p* < 0.05, *p* < 0.001 by two-tailed Student’s t test or one-way ANOVA. Scale bar, 200 μm. anti-p-AMPK (1:1500, Abcam); anti-AMPK (1:1500, Abcam)

We next investigated GDF15’s effect on cellular oxidative stress. ROS measurements using fluorescence imaging revealed distinct patterns of ROS production in AC16 cells under various treatment conditions (Fig. 3A–D, P < 0.05). Gene Set Enrichment Analysis of RNA-seq data from wild-type and GDF15-knockout mice identified specific pathway enrichments (Fig. 3E). Mechanistically, we examined AMPK signaling and found that GDF15 regulated AMPK phosphorylation status in both AC16 cells and primary cardiac myocytes (Fig. 3F–G). In AC16 cells, Dorsomorphin, an AMPK inhibitor, partially reversed GDF15-induced reductions in membrane potential and ROS production (Fig. 3H–K), suggesting that GDF15 modulates ROS generation predominantly through the AMPK signaling pathway.

### Exogenous GDF15 administration attenuates myocardial injury and fibrosis following MI

**Fig. 4:**
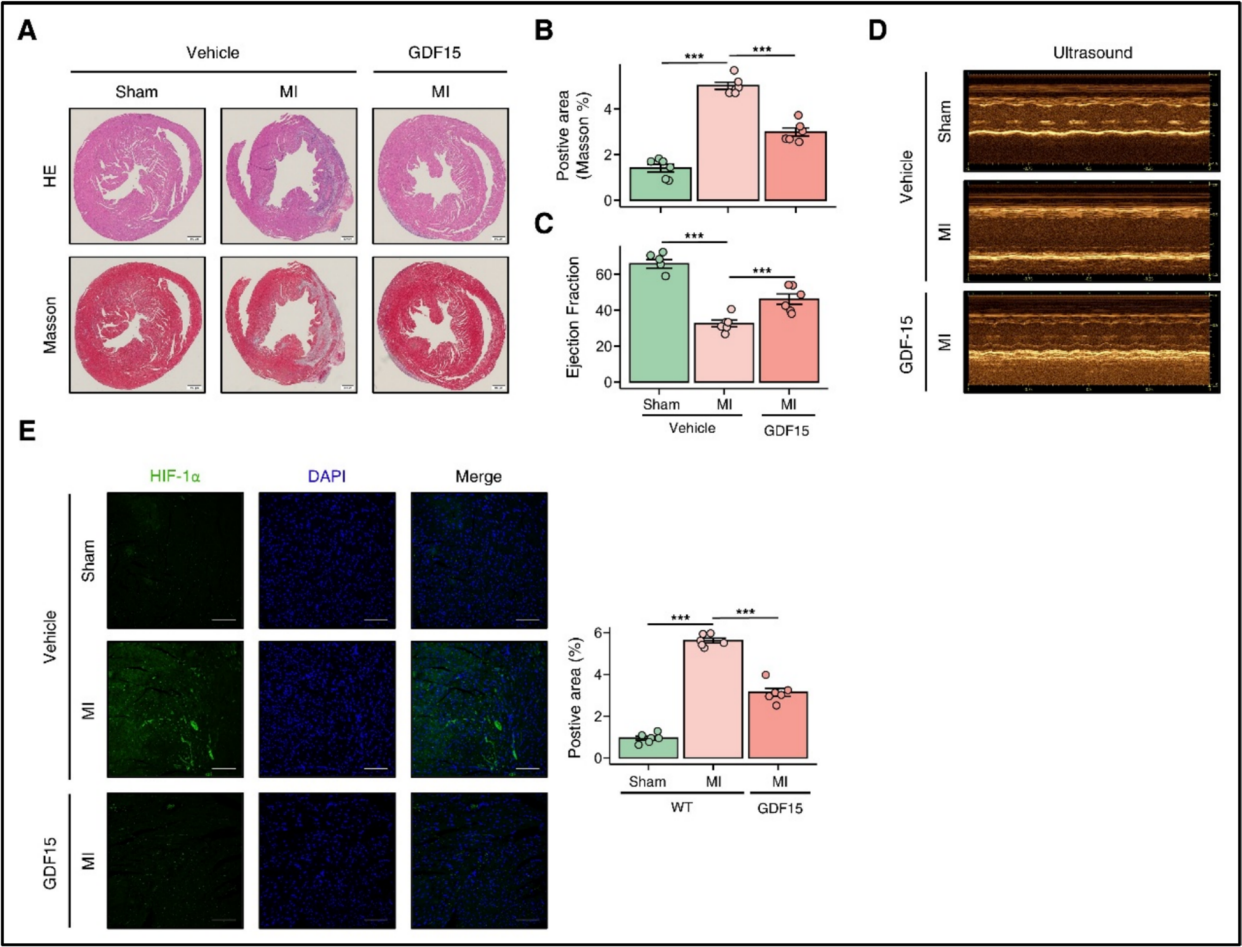
Exogenous Administration of GDF15 alleviates the Progression of Myocardial Infarction. **A**–**B** Representative H&E and Masson staining of cardiac sections from mouse received vehicle or GDF15, with quantification of positive area. **C**–**D** Representative echocardiographic images of hearts from different experimental groups. **E** GDF15 treatment resulted in reduced HIF-1α expression in MI mouse. Data are presented as mean ± SD. n = 6–8/group. *p* < 0.05, *p* < 0.001 by two-tailed Student’s t test or one-way ANOVA. Scale bar, 200 μm. anti-HIF-1α (1:1000, Abcam)

To investigate GDF15’s therapeutic potential in MI, we administered recombinant GDF15 to MI mice. Histological analysis revealed that GDF15 treatment markedly attenuated cardiac inflammation and fibrosis (Fig. 4A–B). Consistent with these histological findings, echocardiographic assessment demonstrated improved cardiac function in GDF15-treated MI mice (Fig. 4C–D p < 0.05). Notably, immunofluorescence analysis showed that GDF15 administration significantly decreased HIF-1α expression in infarcted myocardium (Fig. 4E), suggesting that GDF15 may exert cardioprotective effects partially through modulation of hypoxia-responsive pathways.

## Discussion

MI, a leading cause of mortality and morbidity worldwide, is characterized by sudden coronary occlusion resulting in irreversible myocardial damage [2, 17, 18]. The pathophysiology of MI encompasses complex interactions among ischemia, reperfusion injury, oxidative stress, inflammation, and subsequent fibrosis, collectively contributing to adverse cardiac remodeling and impaired cardiac function [19–21]. Despite advances in reperfusion therapies, MI-associated morbidity and mortality remain substantial, underscoring the urgent need for novel therapeutic strategies targeting underlying mechanisms. Growth differentiation factor 15 (GDF15), a member of the transforming growth factor-β (TGF-β) superfamily, has been implicated in various pathological conditions, including cancer and inflammation [8, 22, 23]. However, its role in MI remains incompletely characterized, creating a critical knowledge gap that this study addresses. Notably, while elevated GDF15 is widely recognized as a powerful prognostic biomarker for adverse outcomes in cardiovascular diseases such as MI [24, 25], a growing body of evidence indicates it also exerts direct cardioprotective effects against insults like pathological hypertrophy [26].

We investigated GDF15’s role in MI through a comprehensive approach combining in vivo and in vitro models. Our investigation elucidated molecular mechanisms by which GDF15 influences cardiac remodeling, mitochondrial function, and oxidative stress regulation following ischemic injury. These findings provide novel insights into GDF15’s cardioprotective effects and its potential as a therapeutic target for MI. Herein, we examine key findings regarding GDF15’s role in myocardial protection, its impact on mitochondrial function and energy metabolism, and its regulatory effects on oxidative stress through the AMP-activated protein kinase (AMPK) signaling pathway. Furthermore, the observed reduction in HIF-1α expression following GDF15 administration underscores its role in mitigating the hypoxic stress integral to myocardial infarction. This finding supports the conclusion that GDF15’s protective effects are, at least in part, mediated through the attenuation of the cellular hypoxic response.

Our study reveals significant insights into molecular mechanisms underlying GDF15’s protective role in MI. Our data demonstrate that GDF15 expression is upregulated following MI, and its absence exacerbates cardiac injury and fibrosis, suggesting a crucial role in mitigating MI-induced damage. Additionally, transcriptional profiling and Western blot analyses revealed that GDF15 deficiency leads to significant enrichment of genes related to mitochondrial respiration, amino acid metabolism, and the AMPK signaling pathway. The reduction in AMPK phosphorylation observed in GDF15-deficient mice indicates that GDF15 may exert protective effects by modulating this critical signaling pathway, which regulates energy metabolism and antioxidant responses. These results elucidate molecular mechanisms by which GDF15 protects the heart while highlighting its therapeutic potential.

At the cellular level, our study demonstrates that GDF15 maintains mitochondrial function and cellular energy metabolism, thereby protecting cardiomyocytes from hypoxic injury. The restoration of mitochondrial membrane potential, cellular oxygen consumption rate, and extracellular acidification rate upon GDF15 supplementation under hypoxic conditions underscores its direct regulatory role on mitochondrial function. These findings are particularly significant as they reveal how GDF15 preserves cellular homeostasis under stress conditions, critical for cardiomyocyte survival during and after MI. Furthermore, the protective effects observed in human cardiomyocyte cell lines suggest that GDF15 may have broad applicability across different cell types, potentially extending its therapeutic relevance to other cardiovascular diseases.

Our results also indicate that GDF15 significantly reduces inflammatory cell infiltration and collagen deposition following MI, thereby mitigating adverse cardiac remodeling. This suggests that GDF15 may exert protective effects, at least partially, through immune response modulation. The observed reduction in fibrosis and inflammation is particularly noteworthy, as these processes significantly contribute to post-MI heart failure progression. GDF15’s ability to suppress inflammatory responses and promote tissue repair enhances our understanding of its role in MI while suggesting potential applications in other inflammatory diseases. Collectively, these findings underscore the multifaceted protective role of GDF15 and its potential as a therapeutic agent for both acute and chronic cardiovascular conditions.

Despite these valuable insights, several limitations warrant acknowledgment. GDF15 has been reported to contribute to disease progression by participating in endoplasmic reticulum stress-related apoptosis [27]. Whether GDF15 similarly mediates cardiomyocyte apoptosis via endoplasmic reticulum stress pathways requires further investigation. Moreover, our findings predominantly derive from mouse models and established cell lines, with absence of absence of primary cells isolated directly from MI mouse model, potentially limiting their ability to recapitulate human cardiac pathophysiology complexity. Clinical validation remains essential to confirm translational potential, and future studies with larger cohorts are warranted to strengthen the robustness of our conclusions.

In summary, our study elucidates GDF15’s multifaceted protective mechanisms in myocardial infarction, including regulation of AMPK signaling, mitochondrial function, reactive oxygen species production, and inflammatory responses. These findings underscore GDF15’s therapeutic potential in mitigating cardiac injury and fibrosis. However, the clinical relevance of these mechanisms requires validation in human studies. Future research should focus on translating these findings into clinical practice, exploring combinatorial therapies, and addressing identified limitations to advance novel cardiac protection strategies.

## Supplementary Information

Supplementary material 1.

Supplementary material 2.

## Data Availability

No datasets were generated or analysed during the current study.
