# Supplementary figures and images for: GDF15 attenuates myocardial infarction-induced injury by preserving mitochondrial function and suppressing oxidative stress
Source: Eur J Med Res. 2025 Sep 29;30:903. doi: 10.1186/s40001-025-03144-8 (PMC12482560; doi:10.1186/s40001-025-03144-8)

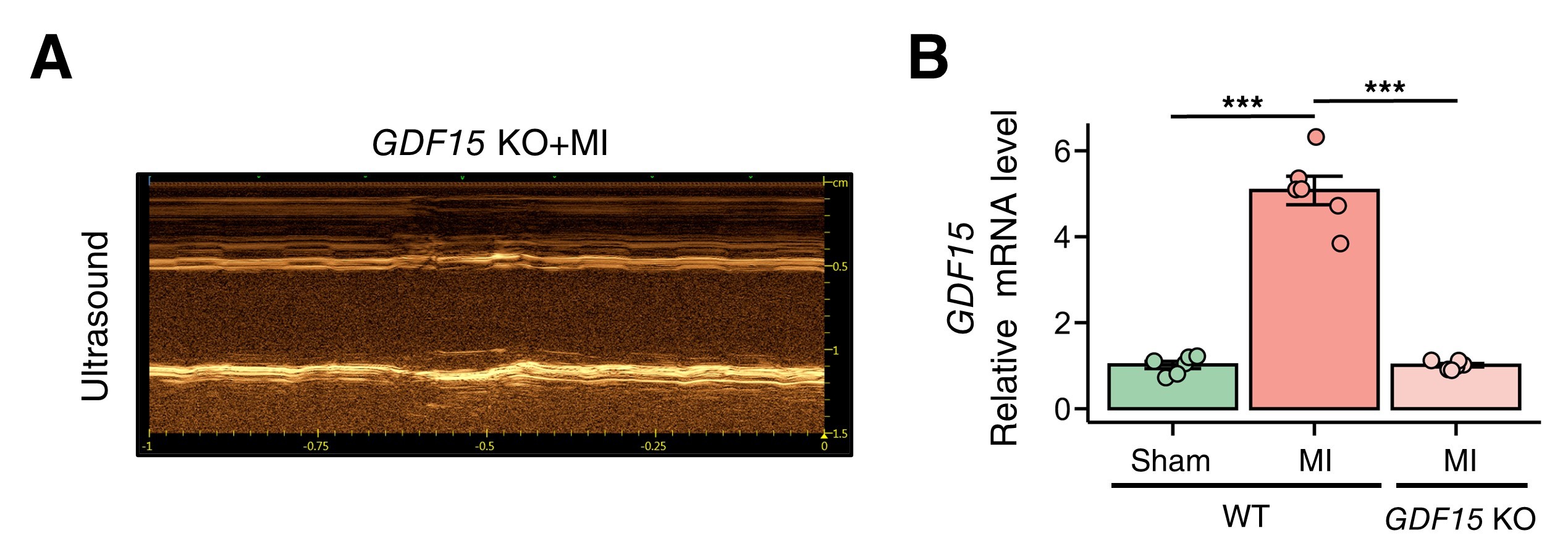

Supplement: Supplementary file 1 — Supplementary material 1. [file 40001_2025_3144_MOESM1_ESM.jpg]
