# Supplementary material for: GDF15 attenuates myocardial infarction-induced injury by preserving mitochondrial function and suppressing oxidative stress
Source: Eur J Med Res. 2025 Sep 29;30:903. doi: 10.1186/s40001-025-03144-8 (PMC12482560; doi:10.1186/s40001-025-03144-8)
Supplement: Supplementary file 2 — Supplementary material 2. [file 40001_2025_3144_MOESM2_ESM.docx]

| **Table S1. ejection fraction (EF) among different experimental mouse groups** | |
| --- | --- |
| Group (n = 6-9) | EF (%) |
| WT (MI) | 44.8±5.9% |
| *GDF15* KO (MI) | 35.6±3.4% |
| WT (MI+GDF-15) | 49.8±4.5% |
